# Study on the Spectrum-Effect Relationship of the Traditional Effect of Saponins in *Glycyrrhiza uralensis* Fisch

**DOI:** 10.1155/2021/6617033

**Published:** 2021-03-16

**Authors:** Wenxin Xia, Qiansong Liu, Hao Zhou, Shiyao Hua, Lin Dong, Xuebo Han, Xueyan Fu

**Affiliations:** ^1^School of Pharmacy, Ningxia Medical University, Yinchuan 750004, China; ^2^Ningxia Medical University, Yinchuan 750004, China

## Abstract

Licorice is a traditional Chinese medicine that has been used for a long time in China and still in great use today. The effect of licorice on tonifying spleen and invigorating qi has been proved for thousands of years, but the material basis of its effect is not clear. In this paper, we established the fingerprints of 21 batches of licorice collected from different origins in China with High-Performance Liquid Chromatography (HPLC) to identify the common peaks. Its effect of tonifying spleen and invigorating qi was confirmed through a series of praxiology experiments. The spectrum-effect relationship between HPLC fingerprints and its effect of tonifying spleen and invigorating qi of licorice was examined by gray relational analysis and partial least squares regression analysis. Results showed that the effect of licorice on tonifying spleen and invigorating qi resulted from various compounds and peaks. *X*_2_–*X*_6_ is presumed to be the main pharmacological substance base. This research successfully identified the spectrum-effect relationship between HPLC fingerprints and the effect of licorice on tonifying spleen and invigorating qi. The research method based on the spectrum-effect relationship helps provide new research ideas and strategies for the study of the basis of the medicinal materials and quality control of traditional Chinese medicine.

## 1. Introduction

*Glycyrrhiza uralensis* Fisch., known as licorice, is one of the most famous traditional Chinese medicine (TCM). Licorice is an “essential herbal medicine” in China; it is not only used to treat diseases. Still, it has been listed as a medicinal and edible homologous plant by the Ministry of Health of China [1]. According to ancient records, licorice has the functions of clearing away heat and detoxification, relieving cough and expectorating phlegm, tonifying spleen and invigorating qi, delaying and relieving pain, and harmonizing all kinds of medicine. These traditional effects have been confirmed by modern research [2]. Triterpenoid saponin is one of the main active components of licorice, which has antiviral, antitumor, immunomodulatory, and other pharmacological effects [3, 4]. In Chinese Pharmacopoeia, the content of Glycyrrhizic Acid and Liquiritin is used as the quality control standard [5]; such a single measurement index is challenging to meet the current quality control requirements of licorice, so the quality standard of licorice still needs to be improved [6].

Due to the characteristics of “multicomponent, multitarget, and comprehensive regulation” of TCM, it is not easy to find and accurately identify the main efficacy components [7]. Meanwhile, it is one-sided to take the content of individual components in Chinese medicine as the standard of quality control. Because of its microscopic, integral, and fuzzy characteristics, fingerprint can better reflect the types and contents of chemical components. Also, the fingerprint has the attributes of integrity, can comprehensively reflect the quality of TCM, has become one of the hot spots in the quality evaluation and control of TCM [8, 9].

Although the fingerprint confirms the consistency of TCM, it does not reflect its clinical efficacy. It is necessary to study the relationship between spectral effect to elucidate the relationship between chemical composition and curative effect [10, 11]. The study of spectrum-effect is a new scientific research method, including the establishment of fingerprint, efficacy evaluation, and correlation analysis of spectrum-effect [12].

So far, researchers have conducted a lot of experimental exploration on the spectrum-effect relationship. At present, the most commonly used method is still to establish fingerprint by HPLC [13, 14] and then conduct spectrum-effect relationship analysis through PCA, correlation analysis, GRA, regression analysis, and other data processing methods. Using modern analytical technology is an essential means to realize the separation of chemical components and identify structure in the fingerprint of traditional Chinese medicine. It is also a necessary method for the study of spectrum-effect correlation. In addition to HPLC, GC-MS [15] and a variety of liquid-mass spectrometry technology can also be used to study the spectral effect relationship, such as high-performance liquid chromatography-tandem mass spectrometry (HPLC-MS/MS) [16], high-performance liquid chromatography-evaporative light scattering (HPLC-ELSD) [17], high-performance liquid chromatography-quadruple-rod time-of-flight mass spectrometry (HPLC-Q-TOF-MS) [18], ultraperformance liquid chromatography-quadruple-rod time-of-flight mass spectrometry (UPLC-Q-TOF-MS) [19], etc.

In our study, we collected licorice from 21 habitats and enriched the total saponin of licorice. We established the fingerprint of licorice saponins from 21 places of origin, identified and determined its common peaks using the similarity evaluation system software of TCM chromatographic fingerprint. After that, we studied the spectrum-effect relationship between the fingerprint and the effect of tonifying spleen and replenishing qi on licorice by the gray relation analysis (GRA) and partial least squares regression (PLSR). This study expects to provide a scientific basis for the basic research of pharmacodynamic substances and quality control standards of licorice.

## 2. Materials and Methods

### 2.1. Plant Materials and Reagents

A total of 21 batches (S1–S21) of licorice samples were collected from different areas in 6 provinces of China: Wuzhong, Ningxia (S1, S2, S3, S4), Longde County, Guyuan City, Ningxia (S5, S6), Lanzhou City, Gansu Province (S7, S8), Wuwei City, Gansu Province (S9), Dingxi City, Gansu Province (S10, S11), Qingyang City, Gansu Province (S12), Baiyin City, Gansu Province (S13), Chifeng City, Inner Mongolia (S14, S15), Erdos City, Inner Mongolia (S16), Luliang City, Shanxi Province (S17), Alar City, Xinjiang Province (S18), Yili, Xinjiang Province (S19, S20), Harbin, and Heilongjiang Province (S21). All batches were authenticated as *Glycyrrhiza uralensis* Fisch. by the pharmacognosy department of Ningxia Medical University.

All standards were purchased from Shanghai Yuanye biotechnology Co., LTD. HPLC-grade acetonitrile, anhydrous ethanol, acetic acid, and methanol were obtained from the Tianjin Damao chemical reagent factory. Also, the instrument used: UV-2600 visible - ultraviolet spectrophotometer (Shimazu instrument Co., LTD), high-performance liquid chromatography-quadrupole in-flight mass spectrometry (Agilent 1290 + 6545), Agilent 1260 high-performance liquid chromatography (Agilent technology co. LTD), etc.

### 2.2. Content Determination

#### 2.2.1. Preparation of Glycyrrhizic Acid Standard Curve

Precision measurement of Glycyrrhizic Acid (GA) reference solution 0.5, 1.0, 2.0, 2.5, 3.0 mL in the test tube, and 60°C dry solvents, plus 5% vanillin-ice acetic acid solution 0.4 mL, shake well, add 1.6 mL perchloric acid, shake well, expose it to 55°C water bath reaction for 20 min, take out with cold water flow cooling to room temperature, do not contain reference substance of the solvent. The determination of absorbance should be at 337 nm wavelength, and the absorbance A should be used as the ordinate and Glycyrrhizic Acid concentration (mg/mL) as the abscissa, drawing a standard curve; the regression equation is as follows:

(1) $$\begin{array}{c} Y=0.225x+0.0132\;\left(R^{2}=0.9995\right) \end{array}$$

The linear relationship of Glycyrrhizic Acid between 0.049 mg/mL and 0.294 mg/mL was good.

The solution of total saponins from 21 samples from different places of origin was collected, and the absorbance of total saponins was determined by the method above. The content was calculated by substituting it into the regression equation.

#### 2.2.2. Solution Preparation of Standard and Test Samples

The standard of Glycyrrhizic Acid was precisely weighed and was dissolved in 10 mL volumetric bottles. The Glycyrrhizic Acid control solution with concentrations of 0.98 mg/mL was prepared.

About 30 mg of the enriched GS from 21 regions was taken and placed in a 10 mL volumetric bottle. 70% ethanol was adjusted to scale and weighed. After 30 min of ultrasound, it was weighed and its quality was supplemented.

#### 2.2.3. Method Validation for Content Determination

- Precision: The precision was evaluated by measuring the control solution of Glycyrrhizic Acid five times in a row.
- (ii)Repeatability: The repeatability was assessed by determining the absorbance of 5 parts of the total saponin solution enriched from samples of S1.
- (iii)Recovery rate: 5.0 mL of total saponins enriched from 9 samples of S1 origin were taken and placed in 25 mL volumetric bottles, respectively. The Glycyrrhizin Acid control solutions 2.0, 4.0, and 6.0 mL were added to form low, medium, and high concentration solutions, respectively. Absorbance was measured according to the method under “2.2.1”, and the recovery rate was calculated.

### 2.3. Establishment of Fingerprint

#### 2.3.1. Apparatus and Chromatographic Conditions

Agilent 1260 liquid chromatography system, Agilent ZORBAX SB-C_18_ column (4.6 × 250 mm, 5 meters), the mobile phase was 0.2% glacial acetic acid (A) and acetonitrile (B). Elution conditions: 0∼15 min, 15% (B); 15∼25 min: 15∼20% (B); 25∼70 min: 20∼50% (B); 70∼90 min: 50∼70% (B); 90∼95 min: 70∼15% (B). The detection wavelength was 254 nm and 310 nm, the flow rate was 1 mL • min-1, the column temperature was 30 °C, and the injection volume was 20 *μ*L.

#### 2.3.2. Sample Preparation

About 30 mg of the enriched GS was collected from 21 regions, respectively. In 10 mL volumetric bottles, 70% ethanol was adjusted to scale and weighed. After 30 min of ultrasound, it was weighed and its quality was supplemented. Finally, the sample was filtered by 0.22 *μ*m microporous membrane, and the filtrate was set aside.

#### 2.3.3. Method Validation for HPLC Fingerprint Analysis

  (i)Precision: The precision was determined by analyzing the total saponins enriched from samples of S1 origin six consecutive times.
  (ii)Stability: The sample stability was evaluated by analyzing one sample in 0, 2, 4, 8, 12, 24 h sample determination.
  (iii)Repeatability: The repeatability was assessed by analyzing six independently prepared total saponins enriched from samples of S1.

### 2.4. Qualitative Analysis of Common Peaks in Fingerprints

#### 2.4.1. Apparatus and Chromatographic Conditions

*HPLC chromatographic conditions*. Agilent 1290 Infinity liquid chromatography system, Agilent RRHD sb-c18 column (2.1 × 100 mm, 1.8 livabilities), the flow rate of 0.200 mL • min-1, column temperature of 30°C, amount of injection of 10 livabilities. The mobile phase was 0.2% formic acid (A) and acetonitrile (B). Elution conditions: 0∼2 min, 15∼30% (B); 2∼10 min: 30∼85% (B); 10∼15 min: 85∼90% (B); 15∼20 min: 90∼0% (B).

*MS conditions*. Dual AJS ESI, with positive particle detection, Gas temperature 350°C; Capillary voltage: 3500 V; Taper hole voltage: 40 V; Extraction voltage: 4.0 V; Ion source temperature: 120°C; Desolvent temperature and flow rate: 350°C, 12 L/min; Atomizing gas voltage: 40 psi; Dryer temperature and flow rate: 350 °C and 10 L/min; Fragmentor 130 V, Skimmer 65 V; Quality scan range: 50∼1000 m/z.

#### 2.4.2. Solution Preparation of Standard and Test Samples

Precisely weigh the standard: Liquiritin, Isoliquiritin, Liquirtigenin, isoliquiritigenin, Liquiritin apioside, Isoliquiritin apioside, Neoliquiritin, Neoisoliquiritin, Glabridin.

Licoagrochalcone A, Licoagrochalcone B, Licoagrochalcone C, Formononetin, Glycyrrhizic Acid: add 70% ethanol to dissolve and dilute them to 4.07, 4.07, 3.38, 4.15, 4.00, 3.84, 3.69, 3.08, 3.30, 3.54, 3.08, 1.92, 3.69, 3.69, 3.69 *μ*g/ml mixed solution, ultrasonically dissolved and set aside.

The total saponin solution enriched by S1 samples prepared by “2.3.2” method was diluted by 70% ethanol by 100 times so that its concentration was about 30 *μ*g/mL and then it was stored for standby.

#### 2.4.3. Qualitative Analysis of Common Peaks

The mixed standard and sample solution were injected into the HPLC-Q-TOF/MS according to the chromatographic and mass spectrometric conditions of “2.5.1”, and the chromatogram was recorded. The spectra of the mixed standard were compared with the spectra of the sample solution, and the common peak components were inferred according to the retention time and the characteristics of the fragment ion peak.

### 2.5. Experimental Animals, Groups, and Dosages

Male SPF grade ICR mice, weighing 18∼22 g, were provided by the laboratory animal center of Ningxia Medical University [Animal certificate NO.: SCXK (Ning) 2015-0001]. The animals were kept at room temperature of 22°C ± 2°C, at a relative humidity of 50∼60%, and natural light. The ICR mice involved in each of the following pharmacodynamics experiments were randomly divided into the model group, the normal group, and the 21 licorice root administration groups (total flavonoids and total saponins), with 10 mice in each group.

### 2.6. Experiment on Reinforcing the Spleen to Replenish Qi

In this study, based on the “Experimental Methodology of Pharmacology of TCM” and the literature method, the method of excessive fatigue and indiscipline of diet was adopted to cause spleen deficiency in mice. The mice in the spleen-deficiency model group showed symptoms such as weight loss, tiredness, loose stool, haggard, and chills.

Building after 14 days, continuous gavage for a week (total saponins of licorice of 150 mg/kg, model group and the normal group was given sodium carboxymethyl cellulose aqueous solution) evaluated the effect on fatigue resistance groups of mice, respectively; mice were put into water depth >20 cm, with a temperature of 25 ± 1°C in the bucket; it was left free to swim and we watched the swimming and recorded the time of death.

#### 2.6.1. Tail Suspension Experiment

Continuous dosing week: 150 mg/kg of total saponins of licorice was given to the model group and the normal group was given sodium carboxymethyl cellulose aqueous solution. After the final dose, the mice were coated with medical adhesive 1 cm from the tail and hung upside down on a pole 15 cm off the ground. The animal struggled to overcome abnormal position, but activity after a while will soon appear desperate behavior. The expression of desperate behavior is no longer a struggle, presenting the character of the quiet state. The mice were observed for 6 min and the immobility time of the mice was recorded after 1 min of the experiment beginning.

#### 2.6.2. Forced Swimming Experiments

The drug was administered continuously for one week (150 mg/kg of total saponins of licorice was given to the model group, and the normal group was given sodium carboxymethyl cellulose aqueous solution). After the last dose, the mice were placed in a cylindrical beaker with a diameter of about 17 cm, a water depth of 10 cm, and a water temperature of 25°C. The observation was performed for 6 min, and the accumulated immobility time was recorded within the last 4 min.

### 2.7. Spectrum-Effect Relationship

Correlation analysis was conducted between the common peak area and pharmacodynamic data of HPLC chromatogram of radix Glycyrrhiza saponin components from 21 various regions. SPSS 24.0 and DPS 7.05 were used for processing, and the common peaks significantly correlated with the pharmacodynamics were found.

### 2.8. Statistical Analysis

SPSS 24.0 statistical software was used for statistical data analysis; DPS software was used for grayscale correlation analysis. GraphPad Prism software was used for mapping, experimental data were represented as means ± SD, and one-way ANOVA LSD and Dunnett's T3 (3) statistical method were used for comparison between groups.

## 3. Investigations and Results

### 3.1. Content of the Total Saponins in Licorice

**Figure 1:**
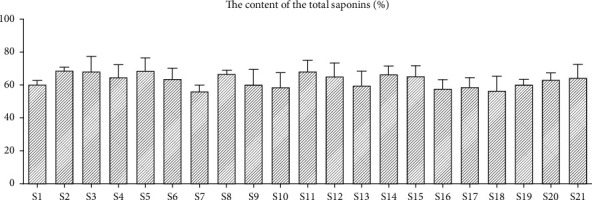
Determination of saponins in *Glycyrrhiza uralensis* Fisch. from 21 producing areas by UV. The result represents mean ± S.D.

The total saponins obtained from 21 samples of origin were collected and the contents of saponins were calculated by Ultraviolet Spectrophotometry (UV). The results showed that more than half of the samples contained more than 60% of total saponins. The specific results are shown in Figure 1.

### 3.2. Analysis of HPLC Fingerprints and Similarities

#### 3.2.1. Method Validation for the HPLC Fingerprint Analysis

The precision, repeatability, and stability of the HPLC fingerprint analysis method were verified. The relative standard deviation (RSD) of the average relative retention time (RRT) and relative peak area (RPA) both meet the prescribed requirements. The variations in the RRT of the characteristic peaks did not exceed 3% and the variations in the RPA did not exceed 5%.

#### 3.2.2. Establishment of the Fingerprint of the Saponins in *Glycyrrhiza uralensis* Fisch

**Figure 2:**
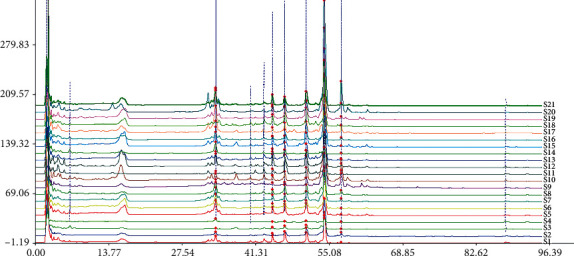
HPLC fingerprint of total saponins of *Glycyrrhiza uralensis* Fisch. from 21 producing areas.

Samples of licorice saponins from 21 different regions were collected, injected into HPLC chromatography, recorded chromatogram, and then imported into the Similarity Evaluation System for Chromatographic Fingerprints of Traditional Chinese Medicine to generate HPLC fingerprints of licorice saponin. The results are shown in Figure 2.

#### 3.2.3. HPLC Fingerprints of Licorice Saponins Samples

**Figure 3:**
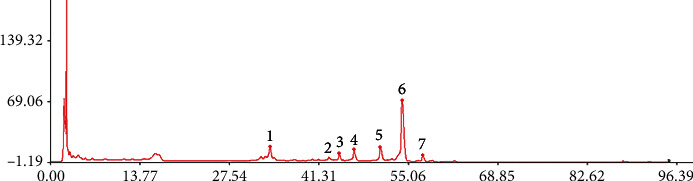
HPLC fingerprint of total saponins of *Glycyrrhiza uralensis* Fisch. from 21 producing areas.

The HPLC fingerprints of the 21 batches of licorice from different areas of China were obtained and matched by the Similarity Evaluation System for Chromatographic Fingerprints of Traditional Chinese Medicine. S1 was selected as the reference spectrum, and the software was corrected by multiple points to automatically match the chromatographic peaks. The median method was selected to generate the control fingerprint, and 7 common peaks were determined. The results are shown in Figure 3.

#### 3.2.4. Similarity Analysis of the HPLC Fingerprints

The similarity value of the generated control fingerprint (R) was set as 1, and the similarity of the characteristic chromatogram of 21 batches of licorice total saponins was calculated, respectively. The similarity between each batch of licorice saponins and the control fingerprint was as follows: 0.929 (S1), 0.991 (S2), 0.916 (S3), 0.805 (S4), 0.944 (S5), 0.895 (S6), 0.973 (S7), 0.804 (S8), 0.932 (S9), 0.898 (S10), 0.915 (S11), 0.901 (S12), 0.930 (S13), 0.931 (S14), 0.951 (S15), 0.766 (S16), 0.753 (S17), 0.916 (S18), 0.961 (S19), 0.911 (S20), 0.954 (S21), which indicates good consistency among the *G. uralensis* samples.

### 3.3. Qualitative Analysis of Common Peaks in HPLC-Q-TOF/MS

HPLC-Q-TOF/MS technology was used to classify the common peak chemical components. According to the above HPLC-Q-TOF/MS analysis conditions, the mixed standard solution and the test solution were, respectively, scanned for positive ions. Based on the cleavage law of licorice compounds in databases such as PubChem and Scifinder, a Qualitative Analysis database of licorice was established by combining the cleavage law and literature reports. Agilent Mass Hunter Qualitative Analysis software was used to analyze the match, combined with the reference, mass spectrometry data in the literature, and chromatographic retention behavior, and finally, the information of the compound was determined. After comparison, four common peaks in fingerprint structures were identified, and the compounds are shown in Table 1.

### 3.4. Results of Reinforcing the Spleen to Replenish Qi

**Figure 4:**
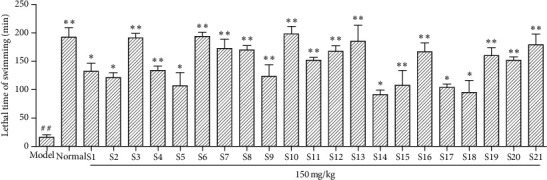
Results of tonifying spleen and tonifying Qi of saponins in *Glycyrrhiza uralensis* Fisch. from 21 producing areas. The result represents mean ± S.D.

As can be seen from the experimental results in Figure 4, compared with the normal group, the swimming time of the model group was significantly reduced ( ^*##*^*P* < 0.01), indicating successful modeling. Compared with the model group, the Glycyrrhiza saponins from S1∼S21 producing area had a significant effect on fatigue-resistant fruit and increased the time of swimming to death ( ^*∗*^*P* < 0.05, ^*∗∗*^*P* < 0.01).

#### 3.4.1. The Results of the Tail Suspension Experiment

**Figure 5:**
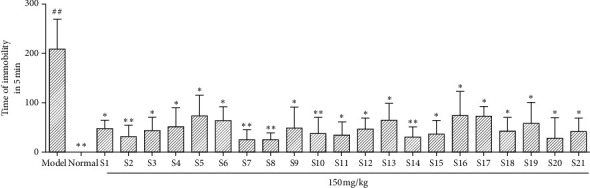
Results of saponins from *Glycyrrhiza uralensis* Fisch. from 21 habitats suspended from the tail of mice. The result represents mean ± S.D. Note: Compared with the normal group ratio,  ^*##*^*P* < 0.01 was an extremely significant difference. Compared with the model group,  ^*∗*^*P* < 0.05*P* < 0.05 means there is a significant difference, ^*∗∗*^*P* < 0.01*P* < 0.01, with an extremely significant difference (*n* = 10).

As can be seen from the experimental results in Figure 5, in the suspended tail experiment, compared with the model group, there were significant differences among the 21 producing areas ( ^*∗*^*P* < 0.05, ^*∗∗*^*P* < 0.01).

#### 3.4.2. The Results of the Forced Swimming Experiment

**Figure 6:**
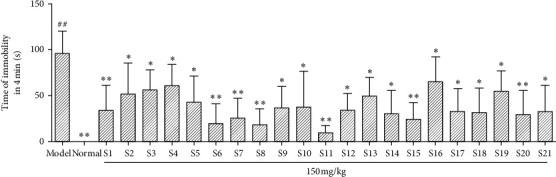
Results of forced swimming experiment of saponins in *Glycyrrhiza uralensis* Fisch. from 21 producing areas. The result represents means ± S.D. Note: Compared with the normal group ratio,  ^*##*^*P* < 0.01 was an extremely significant difference. Compared with the model group,  ^*∗*^*P* < 0.05 means there is a significant difference, ^*∗∗*^*P* < 0.01, with extremely significant difference (*n* = 10).

The mice in the spleen-deficiency model group showed symptoms such as weight loss, tiredness, loose stool, haggard, and chills. The general observation of mice in the treatment group was significantly better than that in the model group. In addition, the antifatigue ability of mice in the treatment group was significantly improved in the forced swimming test. As can be seen from the experimental results in Figure 6, in the forced swimming experiment, compared with the model group, there were significant differences among the 21 producing areas ( ^*∗*^*P* < 0.05, ^*∗∗*^*P* < 0.01).

### 3.5. Analysis of the Spectrum-Effect Relationship

Total saponins parts of tonifying spleen replenishing qi experiment, forced swimming test, and tail suspension test have obvious effect. Therefore, correlation analysis was carried out between the common peak area of the licorice saponins in the HPLC chromatogram of each locality and the pharmacodynamic data of the effective invigorating spleen and invigorating qi experiment, forced swimming experiment, and suspended tail experiment.

#### 3.5.1. Gray Relation Analysis (GRA) Results

Gray relational analysis is a method to describe the size, strength, and order of the factors by using gray relational analysis. In this research, the common peak area of the fingerprint of licorice saponins from 21 places of origin was taken as a group of variables, and the data of the pharmacodynamics experiment was taken as another group of variables. The common peak significantly related to the efficacy was found through DPS 7.05 software gray correlation analysis.

It can be seen from Table 2 that there were 6 peaks with the correlation degree of fatigue resistance and swimming time greater than 0.5 in the experiment of invigorating spleen and replenishing qi, namely, common peaks *X*_5_, *X*_2_, *X*_4_, *X*_1_, *X*_6_, and *X*_3_. It indicated that the above peaks were closely related to the efficacy of medicine and might contribute more to the efficacy of invigorating the spleen and replenishing qi. The correlation degree of peak 7 was less than 0.5, indicating that this compound may contribute little to the effect of invigorating the spleen and replenishing qi.

It can be seen from Table 3 that there were 6 peaks whose correlation degree with the cumulative fixed time of the suspended tail experiment was greater than 0.5, namely, common peaks *X*_6_, *X*_2_, *X*_4_, *X*_1_, *X*_5_, *X*_7_, and *X*_3_. It indicated that the above peaks were closely related to the efficacy of drugs.

It can be seen from Table 4 that there were 5 peaks whose correlation degree with the cumulative fixed time of the forced swimming experiment was greater than 0.5, indicating that the 5 common peaks mentioned above were closely related to the drug effect. The correlation degree of peak *X*_1_ and peak *X*_7_ is less than 0.5, indicating that these two compounds might not play the role of fatigue resistance.

#### 3.5.2. PLSR Analysis Results

Partial least squares regression analysis (PLSR) integrates multiple functions to realize regression modeling, data structure simplification, and correlation analysis between two sets of variables at the same time. It can make maximum use of data information and has the characteristics of high prediction accuracy and easy model interpretation. Through DPS software and PLSR analysis, the peak area of fingerprints of licorice saponins from 21 places was taken as an independent variable (*X*), and the efficacy data obtained from each experiment was taken as dependent variable (*Y*) for PLS regression analysis to obtain the regression equation fitted by *X* and *Y*. The regression coefficient reflects the contribution of each *X* to *Y*. The greater the absolute value of the PLS regression coefficient, the greater the contribution of the common chromatographic peak will be.

*Experiment on tonifying spleen and replenishing Qi*. By PLSR analysis, the relevant regression equation was fitted as follows:

(2) $$\begin{array}{c} Y=-0.2178X_{1}+0.0607X_{2}+0.1084X_{3}-0.1177X_{4}-0.0018X_{5}+0.1266X_{6}+0.1508X_{7} \end{array}$$

In this regression equation, the contribution rates of the seven common peaks were: *X*_1_ *>* *X*_7_ *>* *X*_6_ *X*_4_ *>* *X*_3_ *>* *X*_2_ *>* *X*_5_ in turn. In the fingerprint, the peak numbers *X*_1_, *X*_4_, and *X*_5_ were negatively correlated with the swimming time. That is, the peak strength of this chromatographic peak was increased, and the corresponding swimming time might be reduced, while the remaining chromatographic peaks were positively correlated.

*Tail suspension test*. By PLSR analysis, the relevant regression equation was fitted as follows:

(3) $$\begin{array}{c} Y=-0.0715X_{1}+0.0624X_{2}-0.0401X_{3}+0.0943X_{4}+0.1370X_{5}+0.0248X_{6}-0.0977X_{7} \end{array}$$

In this regression equation, the contribution rates of the seven common peaks were *X*_5_ > *X*_7_ > *X*_4_ > *X*_1_ > *X*_2_ > *X*_3_ > *X*_6_ in turn. In fingerprints, peaks *X*_1_, *X*_3_, and *X*_7_ were negatively correlated with the accumulative immobility time. That is, the peak strength of this chromatographic peak increased, the corresponding immobility time might decrease, and the remaining chromatographic peaks were positively correlated.

*Forced swimming experiment*. By PLSR analysis, the relevant regression equation was fitted as follows:

(4) $$\begin{array}{c} Y=-0.1047X_{1}-0.0980X_{2}-0.0660X_{3}-0.1469X_{4}-0.1101X_{5}-0.0704X_{6}-0.0460X_{7} \end{array}$$

In this regression equation, the contribution rates of the seven common peaks were *X*_4_ > *X*_5_ > *X*_1_ > *X*_2_ > *X*_6_ > *X*_3_ > *X*_7_ in turn. But in the fingerprint, peaks *X*_1_, *X*_2_, *X*_3_, *X*_4_, *X*_5_, *X*_6_, and *X*_7_ were negatively correlated with the accumulated immobility time. That is, the peak strength of this chromatographic peak was increased; the corresponding immobility time might be reduced.

## 4. Discussion

Traditional Chinese medicine (TCM) has been welcomed worldwide because of its long history and good curative effect. However, up to now, the quality control standard of TCM still needs further discussion and improvement [20, 21]. In recent years, there are various analytical methods for quality control of traditional Chinese medicine, including biological chromatography, traditional chromatography, DNA method, Fourier infrared, near-infrared, nuclear magnetic resonance, and other spectral methods [22]. However, the existing quality control standards that take the characteristics of a single or multiple chemical components as the standard seem unreasonable [23]. Therefore, it is necessary to develop a reasonable method to find markers for quality control of TCM. Thus, the study of the relationship between fingerprint and efficacy emerged [24]. The study of the spectrum-effect relationship is of great significance to elucidate the active components and the quality control of TCM.

In the present study, a simple, accurate, and validated chromatographic fingerprinting method was developed and used to analyze 21 batches of licorice saponins samples collected from different regions in China. Firstly, the total saponins of licorice were prepared. 1000 g of licorice herbs from 21 producing areas was weighed and decocted with five times of water, 3 times in total, 1 h each time. The combined filtrate was left standing at room temperature; 60% alcohol was added and left overnight. The supernatant was filtrated, and the combined filtrate was decompressed and recovered until the extracts of licorice herbs were alcohol-free. The extracts of licorice were, respectively, passed into an 80–100 mesh polyamide chromatography column and then eluted with distilled water, 30% ethanol, and 70% ethanol successively. Each component was eluted until the color became lighter and colorless. The eluent was concentrated under pressure and finally concentrated in three parts with different polarities in each producing area, namely water part, 30% alcohol part, and 70% alcohol part. The eluent of the water part was decompressed and evaporated to get the total saponins of licorice and stored for later use. Second, HPLC fingerprint was established, which is a common method in the quality evaluation of traditional Chinese medicine. The consistency of traditional Chinese medicine was evaluated more comprehensively than the conventional way [25]. HPLC fingerprint and HPLC-QTOF-MS methods used in the present study are important techniques for quality evaluation and identification of natural products [26, 27], and they are more and more widely used in the research of quality evaluation and control of natural products including herbal medicine. Then, 7 common peaks were found by fingerprint similarity evaluation software, and 4 peaks were identified by HPLC-Q-TOF/MS. Second, we conducted a modern pharmacodynamic study of the traditional effects of licorice. It can be seen from the efficacy test results that the total saponins of licorice root have obvious effects on the spleen and qi strengthening test, forced swimming test, and tail suspension test, indicating that the saponins may be the main material basis of these effects. Finally, the relationship between its biological activity and HPLC fingerprint was analyzed by GRA and PLSR. GRA is based on the similarity of geometric curves of various factors to determine the correlation between the factors [28]. If the trend of differences between two factors is consistent, the two factors are considered strongly related. Regression analysis is a kind of multivariate statistical method, which uses regression equation to study the dependence relationship between multiple variables. The contribution of a drug to pharmacodynamics can be determined by establishing a regression equation of fingerprint peak and pharmacodynamics data. PLSR is one of the regression analysis methods [29].

The experimental results identified a total of 7 common peaks (*X*_1_, *X*_2_, *X*_3_, *X*_4_, *X*_5_, *X*_6_, *X*_7_) and inferred 4 common peaks, namely *X*_4_ (Glabrolide), *X*_5_ (Stearyl Glycyrrhetate), *X*6 (Glycyrrhizic Acid), and *X*_7_ (18*α*-Glycyrrhizic Acid). *X*_4_, *X*_5_, and *X*_6_ all had a high contribution in three experiments of tonifying spleen and invigorating qi, suspended tail, and forced swimming. We can speculate that these three compounds may be the active ingredients to exert the above effects.

There are still some deficiencies in the experimental methods of this study. At first, the enriched GS is usually extracted and prepared in ethanol by sonication, and it may not be the most effective method for extracting active components from the *Glycyrrhiza uralensis*. The emerging potential methods of pretreatment and extraction of active components [30] may bring unexpected insights into the material basis of *Glycyrrhiza uralensis*. Then, there is no reverse validation of the predicted active components, which can further determine the scientific nature of the spectral efficiency relationship. The study of the spectrum-effect relationship can not only effectively identify different sources of *Glycyrrhiza uralensis* but also reveal the material basis of its efficacy. Also, it can provide some scientific basis for improving quality control standards.

## Figures and Tables

**Table 1:** Assignment of Common peaks in the fingerprint of saponins in *Glycyrrhiza uralensis* Fisch. based on HPLC-Q-TOF/MS.

Peak number	Name	Formula	RT	*m*/*z*	Mass	Theoretical isotopic
4	Glabrolide	C_30_H_44_O_4_	5.990	468.6679	467.6234	468.3240
5	Stearyl Glycyrrhetate	C_48_H_82_O_4_	8.862	724.1758	723.1622	723.6347
6	Glycyrrhizic Acid	C_42_H_62_O_16_	11.308	845.4114	822.4116	822.4038
7	18*α*-Glycyrrhizic Acid	C_42_H_62_O_16_	12.315	845.3916	822.4019	823.4071

**Table 2:** Correlation between the common peak of saponins from Glycyrrhiza uralensis Fisch. and the effect of tonifying spleen and replenishing qi.

No.	Correlation	No.	Correlation
1	0.5970	5	0.5960
2	0.6605	6	0.6670
3	0.5806	7	0.5846
4	0.6475	—	—

**Table 3:** Correlation between common peaks of saponins from *Glycyrrhiza uralensis* Fisch. and tail suspension test.

No.	Correlation	No.	Correlation
1	0.5970	5	0.5960
2	0.6605	6	0.6670
3	0.5806	7	0.5846
4	0.6475	—	—

**Table 4:** Correlation between common peaks of saponins from *Glycyrrhiza uralensis* Fisch. and forced Swimming experiment.

No.	Correlation	No.	Correlation
1	0.5281	5	0.5937
2	0.6035	6	0.5600
3	0.5821	7	0.4424
4	0.6090	—	—

## Data Availability

The underlying data supporting the study are available within the article.
